# Genome-wide association analysis and admixture mapping in a Puerto Rican cohort supports an Alzheimer disease risk locus on chromosome 12

**DOI:** 10.3389/fnagi.2024.1459796

**Published:** 2024-09-04

**Authors:** Bilcag Akgun, Briseida E. Feliciano-Astacio, Kara L. Hamilton-Nelson, Kyle Scott, Joe Rivero, Larry D. Adams, Jose J. Sanchez, Glenies S. Valladares, Sergio Tejada, Parker L. Bussies, Concepcion Silva-Vergara, Vanessa C. Rodriguez, Pedro R. Mena, Katrina Celis, Patrice G. Whitehead, Michael Prough, Christina Kosanovic, Derek J. Van Booven, Michael A. Schmidt, Heriberto Acosta, Anthony J. Griswold, Clifton L. Dalgard, Katalina F. McInerney, Gary W. Beecham, Michael L. Cuccaro, Jeffery M. Vance, Margaret A. Pericak-Vance, Farid Rajabli

**Affiliations:** ^1^John P. Hussman Institute for Human Genomics, University of Miami Miller School of Medicine, Miami, FL, United States; ^2^Universidad Central del Caribe, Bayamón, PR, United States; ^3^Clinica de la Memoria, San Juan, PR, United States; ^4^Dr. John T. Macdonald Foundation Department of Human Genetics, University of Miami Miller School of Medicine, Miami, FL, United States; ^5^Department of Anatomy, Physiology, and Genetics, Uniformed Services University of the Health Sciences, Bethesda, MD, United States; ^6^Department of Neurology, University of Miami Miller School of Medicine, Miami, FL, United States

**Keywords:** Alzheimer disease, chromosome 12, genome-wide association study, admixture mapping, Puerto Ricans

## Abstract

**Introduction:**

Hispanic/Latino populations are underrepresented in Alzheimer Disease (AD) genetic studies. Puerto Ricans (PR), a three-way admixed (European, African, and Amerindian) population is the second-largest Hispanic group in the continental US. We aimed to conduct a genome-wide association study (GWAS) and comprehensive analyses to identify novel AD susceptibility loci and characterize known AD genetic risk loci in the PR population.

**Materials and methods:**

Our study included Whole Genome Sequencing (WGS) and phenotype data from 648 PR individuals (345 AD, 303 cognitively unimpaired). We used a generalized linear-mixed model adjusting for sex, age, population substructure, and genetic relationship matrix. To infer local ancestry, we merged the dataset with the HGDP/1000G reference panel. Subsequently, we conducted univariate admixture mapping (AM) analysis.

**Results:**

We identified suggestive signals within the *SLC38A1* and *SCN8A* genes on chromosome 12q13. This region overlaps with an area of linkage of AD in previous studies (12q13) in independent data sets further supporting. Univariate African AM analysis identified one suggestive ancestral block (*p* = 7.2×10^−6^) located in the same region. The ancestry-aware approach showed that this region has both European and African ancestral backgrounds and both contributing to the risk in this region. We also replicated 11 different known AD loci -including *APOE*- identified in mostly European studies, which is likely due to the high European background of the PR population.

**Conclusion:**

PR GWAS and AM analysis identified a suggestive AD risk locus on chromosome 12, which includes the *SLC38A1* and *SCN8A* genes. Our findings demonstrate the importance of designing GWAS and ancestry-aware approaches and including underrepresented populations in genetic studies of AD.

## 1 Introduction

Alzheimer Disease (AD), the most common type of dementia in older adults worldwide, accounts for an estimated more than 60% of all dementia cases (Alzheimer’s Association, 2024). The prevalence of AD increases with age, affecting more than a third of individuals above the age of 85 (Borenstein and Mortimer, 2016). The etiology of AD is complex with a strong genetic predisposition (Gatz et al., 1997; Gatz et al., 2006). Genome-wide association studies (GWAS) have identified more than 75 loci associated with AD to date (Bellenguez et al., 2022). However, these studies have primarily focused on non-Hispanic White (NHW) populations (Mills and Rahal, 2019; Mills and Rahal, 2020). Research into AD genetics across diverse populations reveals a partial overlap of genetic risk and protective loci among different ancestral groups, while also showing differences in effect sizes and specific genetic variants associated with AD (Cukier et al., 2016; Farrer et al., 1997; Liu et al., 2009; Reitz et al., 2013). Including diverse populations in AD genetic studies is crucial for identifying ancestry-specific loci and generalizing risk and protective loci across ancestral populations (Reitz et al., 2023). Notably, Latino populations are among the least represented in AD genetic studies (Mills and Rahal, 2020), underscoring the necessity of extending AD genetic studies to these populations, particularly given their admixed ancestral makeup. This is essential for a more comprehensive understanding of the genetic architecture of AD and advancing the development of precision medicine.

The diverse and multicultural Puerto Rican (PR) population is the second largest Latino group in the continental US. The estimated AD prevalence among PRs is 12.5%, which is higher compared to the general US population (10.1%) (Feliciano-Astacio et al., 2019). The PR population is three-way admixed with an average of 69% of European (EU), 17% African (AF) and 14% Amerindian (AI) ancestral backgrounds (Feliciano-Astacio et al., 2019). The admixed background in the PR population facilitates the discovery of novel AD loci and allows for the assessment of heterogeneity in the effects of known AD loci across EU, AF and AI ancestral backgrounds. However, genetic studies on PRs for AD have been limited so far.

To address these issues, we performed GWAS, ancestry-aware approaches, and comprehensive analyses to identify novel AD susceptibility loci and characterize known AD genetic risk loci and regions in PR individuals enrolled in AD genetic studies.

## 2 Materials and methods

### 2.1 Study participants

**Figure 1:**
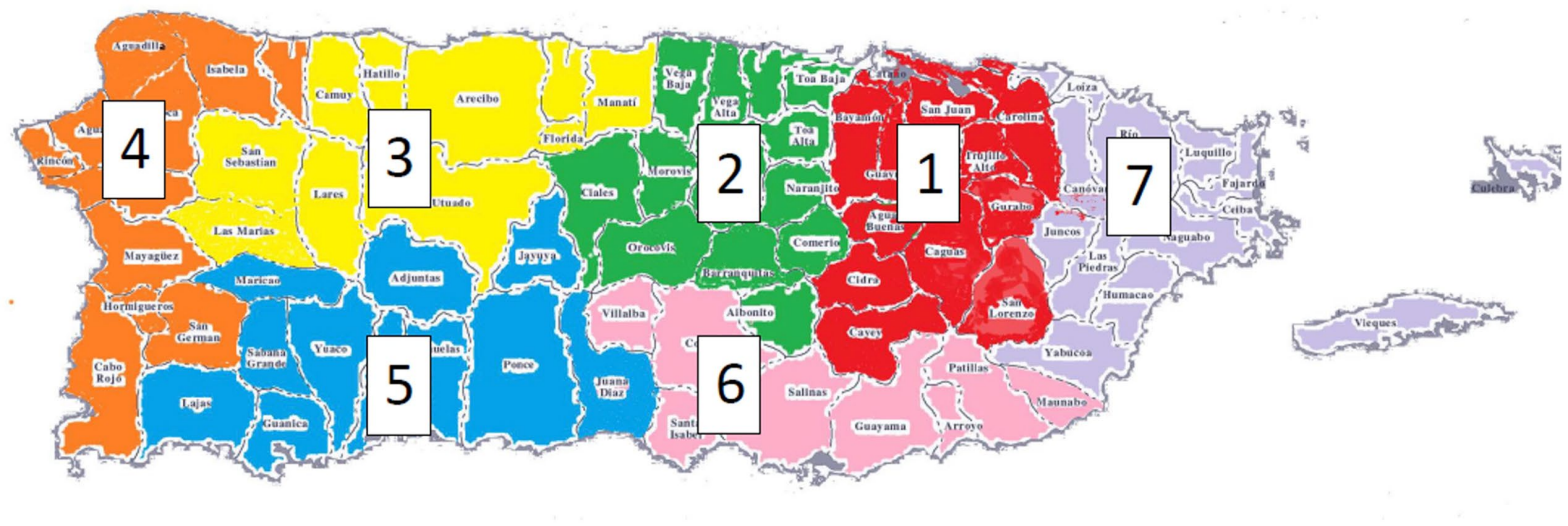
Seven different geographical Puerto Rican health zones defined by Puerto Rico Department of Health (2024) where participants were ascertained.

The participants were ascertained from seven different regions of Puerto Rico (94%) (Figure 1), and from the continental United States (6%) (Florida, New York, Connecticut, and North Carolina). All ascertainment was coordinated by the University of Miami and the Universidad Central del Caribe.

Informed consent was obtained from all participants, and the study protocols were approved by the University of Miami’s, and the Universidad Central del Caribe’s Institutional Review Boards. All eligible participants underwent an initial screening consisting of a standard clinical interview which included detailed medical and family history as well as a Modified Mini-Mental State Examination (3MS) (Folstein et al., 1975; Teng and Chui, 1987). Individuals who failed the screening were then evaluated with a comprehensive multi-domain cognitive battery which included measures of memory, executive function, language, and visuospatial ability. In addition, these participants were evaluated using functional measures including the Clinical Dementia Rating Scale (CDR). Using all available clinical information, participants were adjudicated by neurologists and neuropsychologists with expertise in neurodegenerative disorders. Clinical research diagnoses were assigned using the National Institute of Aging-Alzheimer’s Association (NIA-AA) criteria for possible and probable AD (McKhann et al., 2011) or the DSM-V criteria for Major Neurocognitive Disorder, Alzheimer’s type (Association AP, 2013). AD Cases were defined as participants who met NIA-AA or DSM-V criteria for AD. In summary, possible and probable AD diagnoses were assigned using the NIA-AA criteria by a clinical adjudication panel after reviewing historical and screening/evaluation test data (Rajabli et al., 2018; Rajabli et al., 2021). Cognitively unimpaired (CU) individuals were defined as participants who were cognitively unimpaired and ≥ 65 years of age at study entry.

### 2.2 Whole genome sequencing

Whole genome sequencing (WGS) data was generated at the Uniformed Services University of the Health Sciences (USUHS) and the Center for Genome Technology (CGT) at the John P. Hussman Institute for Human Genomics (HIHG) at the University of Miami Miller School of Medicine using coordinated methodology. Briefly, sequencing libraries were created using the TruSeq DNA PCR-Free library preparation kit followed by sequencing to 30X depth on the Illumina NovaSeq 6000 (Illumina, San Francisco, California, United States). The resulting FASTQ files were processed on a high-performance computing cluster maintained by the Frost Institute for Data Science and Computing at the University of Miami. Processing and quality control utilized the Variant Calling Pipeline (VCPA) developed and used for the Alzheimer’s Disease Sequencing Project (Leung et al., 2019) including alignment to GRCh38 using bwa-mem (Li, 2013), duplicates marking and base quality recalibration with multi-sample variant calling and joint genotyping were performed using the GATK HaplotypeCaller (van der Auwera and O’Connor, 2020) across all samples from the study. After quality control, all samples were screened for causal variants of *PSEN1, PSEN2* and *APP* genes, and individuals who were found to be carriers of any causal variant were excluded from the study.

Principal components (PCs) were calculated using the GENESIS R/Bioconductor package (Gogarten et al., 2019). To determine the PCs used for further analyses, we employed logistic regression modelling (AD ~ Sex + Age + PC1:10).

### 2.3 Association analysis

#### 2.3.1 Single variant analysis

Single variant association analysis was performed using SAIGE (Zhou et al., 2020) on genotypes employing a linear mixed model. We analyzed the data in two separate models; the first model accounted for sex, age, and PCs for population substructure (Model 1), while the second model also included the dosage of the *APOE ε4* allele (Model 2). In both models, we included a genetic relationship matrix as a random effect to account for any potential relatedness. The GenABEL package version 1.8–031 was used to estimate genomic inflation (λ). Known AD markers were determined from the AF (Kunkle et al., 2021) and NHW (Bellenguez et al., 2022) GWASs. We evaluated whether these Known AD markers were replicated in our association analysis results for both models based on the *p*-value threshold of 0.05.

#### 2.3.2 Gene-based analysis

Before the gene-based test, variants were restricted to rare variants excluding all variants with minor allele frequency (MAF) > 0.01. Then, variants were annotated with AnnoVar (Wang et al., 2010) to identify the gene region and the CADD (Kircher et al., 2014) score. As a result of gene region annotation, only intragenic variants (upstream, downstream, exonic and intronic variants) were included in the analysis. A combined test of burden and sequence kernel association test (SKAT-O) (Lee et al., 2012) was performed using the SAIGE-GENE (Zhou et al., 2020) tool. Three different variant sets were assessed: CADD20 set (variants with a CADD score of 20 or higher), CADD10 set (variants with a CADD score of 10 or higher) and CADD0 set (all intragenic variants). All sets were tested twice with two models: a main model (adjusted for sex, age, and first 4 PCs as fixed effects and GRM as a random effect), and an additional *APOE ε4* allele dosage adjusted model.

### 2.4 Pathway analysis

We performed pathway analyses with Multi-marker Analysis of GenoMic Annotation (MAGMA) (de Leeuw et al., 2015) v1.08 using FUMA (Watanabe et al., 2017) v1.5.6, which performs SNP-wise gene analysis. 18977 gene sets obtained from MsigDB (Liberzon et al., 2015; Subramanian et al., 2005) v7.0 were used in the analyses. We analyzed a 35-kb upstream and 10-kb downstream window around each gene.

### 2.5 Fine-mapping and ancestral aware analysis

#### 2.5.1 Fine mapping and replication analysis

Fine-mapping was performed using CARMA (Yang et al., 2023) with each locus defined as a 1Mb region centered around the index SNP with suggestive significant (*p* < 1×10^−6^) loci. Each locus’ LD matrix was generated based on the individual-level genetic data used in the association analysis. We employed CARMA with default values for all parameters with the maximum number of causal variants assumed in a region set at *N* = 10. The functional annotation CADD (Kircher et al., 2014) was also provided to CARMA as prior information on the causality of the testing SNPs.

For replication analysis, we used the EFIGA (Estudio Familiar de Influencia Genetica en Alzheimer) (Vardarajan et al., 2014) cohort included in the ADSP R4 dataset. This cohort includes individuals of Caribbean Hispanic descent recruited from the Dominican Republic and New York, comprising both a family-based study with multiple AD individuals and a case–control study of unrelated AD individuals. We selected AD cases and CU controls with ≥65 years of age at study entry from this cohort. PCs were calculated and single variant association testing was performed on index SNPs at suggestive significant loci identified in our PR dataset, employing the same statistical models, tools, and adjustments as in the initial analysis. We then conducted a meta-analysis of these suggestive index variants across the PR and EFIGA datasets using the METASOFT (Han and Eskin, 2011) program with random effects model (RE2).

#### 2.5.2 Global ancestry estimation

The admixture proportion was estimated by using a model-based clustering algorithm implemented in the ADMIXTURE software (Zhou et al., 2011). Supervised ADMIXTURE analysis was performed at K = 3 by including the 3 reference populations (AI, EU, and AF) from combined reference panels of the Human Genome Diversity Project (HGDP) (Fairley et al., 2020) and 1000 Genomes Phase 3 (Delaneau et al., 2014; Auton et al., 2015).

#### 2.5.3 Local ancestry estimation

The local ancestry was assessed by combining the 3 populations (AI, EU, and AF) in combined reference panels of HGDP (Fairley et al., 2020) and 1000 Genomes Phase 3 (Delaneau et al., 2014; Auton et al., 2015) with the PR dataset. The SHAPEIT (Delaneau et al., 2011) tool was used to phase all individuals in the same combined reference panels, and the RFMix Version 2 (Maples et al., 2013) tool with the discriminative modelling approach was used to infer the local ancestry at each locus across the genome. The standard parameters were used with a minimum node size of 5 to perform RFMix analysis.

#### 2.5.4 Admixture mapping

We performed admixture mapping in PR datasets using the GENESIS R/Bioconductor package (Gogarten et al., 2019). First, we encoded copies of local ancestry calls for each ancestry (AF, AI, and EU) as dosage values (0, 1, or 2, number of haplotypes at a locus). Then, to test for an association between AD and local ancestry at a genomic location, we used a logistic mixed model. The model includes local ancestry as the main and the genetic relationship matrix (GRM) as a random effect to adjust for the sample relatedness and was adjusted further for age, sex, and principal components (PC1:4).

### 2.6 Runs of homozygosity analysis

We calculated ROH in the PR dataset by including the 3 reference populations (AI, EU, and AF) from combined reference panels of the Human Genome Diversity Project (HGDP) (Fairley et al., 2020) and 1000 Genomes Phase 3 (Delaneau et al., 2014; Auton et al., 2015) using the PLINK software. The following parameters were used: -homozy-snp 50, -homozy-kb 300, homozy-density 300, homozyg-gap 1000, -homozyg-window-snp 50, -homozyg-window-het 1, homozyg-window-missing 1, and homozyg-window-threshold 0.05. We plotted the resulting outputs using the ggplot package of the R.

We analyzed the total and average lengths of the ROHs per sample and the total number of ROHs for each sample. Then we evaluated ROHs larger than 1 Mb, 2 Mb, or 3 Mb separately with the global burden analysis. We conducted a global burden analysis among autosomal chromosomes in cases and controls using a one-tailed test with 10,000 permutations for the number of ROHs, the total ROH length and the mean ROH length per individual.

### 2.7 Polygenic risk score

We constructed PRS on the PR dataset using the effect sizes from summary statistics from the largest NHW GWAS study (Bellenguez et al., 2022). Quality control steps were carried out using standard parameters in the literature (Choi et al., 2020). We removed duplicate and ambiguous SNPs from the summary statistics NHW GWAS with the custom script.

The PRSice-2 (Choi and O'Reilly, 2019) tool was used to generate the PRS. Analyses were performed with standard parameters in accordance with the published PRS tutorial (Choi et al., 2020). We applied LD-clumping using the following parameters: --clump-kb 250 – clump-r2 0.1 –clump p1. We also filtered out variants with minor allele frequency (MAF) was less than 5%. We included only autosomal chromosomes in the analysis. In order to evaluate PRS performance independent of the *APOE* effect, we first removed the *APOE* region (2 MB around *APOE ε4* SNP) from the data. Then, to adjust the model, we used age, sex, and the first four PCs as covariates.

After each PRS calculation, the PRS performance was assessed by employing the logistic regression model: Covar-only, PRS-only, *APOE ε4*-only, PRS + *APOE ε4*, and Full to construct receiver operator curves (ROC).

1. AD ~ Sex + Age + PC1:4 (“Model_Covar-only_”)
2. AD ~ PRS (“Model_PRS-only_”)
3. AD ~ *APOE ε4* (“Model_*APOE ε4*-only_”)
4. AD ~ PRS + *APOE ε4* (“Model_PRS + *APOE ε4*_,”)
5. AD ~ PRS + *APOE ε4* + Sex + Age + PC1:4 (“Model_Full_”)

We deposited the codes and scripts used in this study to the GitHub repository we created.^1^

## 3 Results

### 3.1 Association analysis

Our study included a total of 648 PR individuals from families (78 AD, 41 cognitively unimpaired) and unrelated individuals (267 AD, 262 cognitively unimpaired) (Table 1). There was no evidence for genomic inflation (model 1: λ = 1.029; model 2: λ = 1.048).

**Table 1:** Table showing the age, gender, and *APOE ε4* dosage distributions of the participants in our study.

		Cases	Controls	Total
Count		345	303	648
	Families	78	41	119
	Unrelated individuals	267	262	529
Sex	Female	240 (69.6%)	225 (74.2%)	465 (71.8%)
	Male	105 (30.4%)	78 (25.8%)	183 (28.2%)
Age (Mean ± SD)		75.5 ± 8.09	75.3 ± 6.74	75.5 ± 7.48
*APOE ε4*%	0	57.7	76.2	66.4
	1	35.6	21.5	29.0
	2	6.7	2.3	4.6

**Figure 2:**
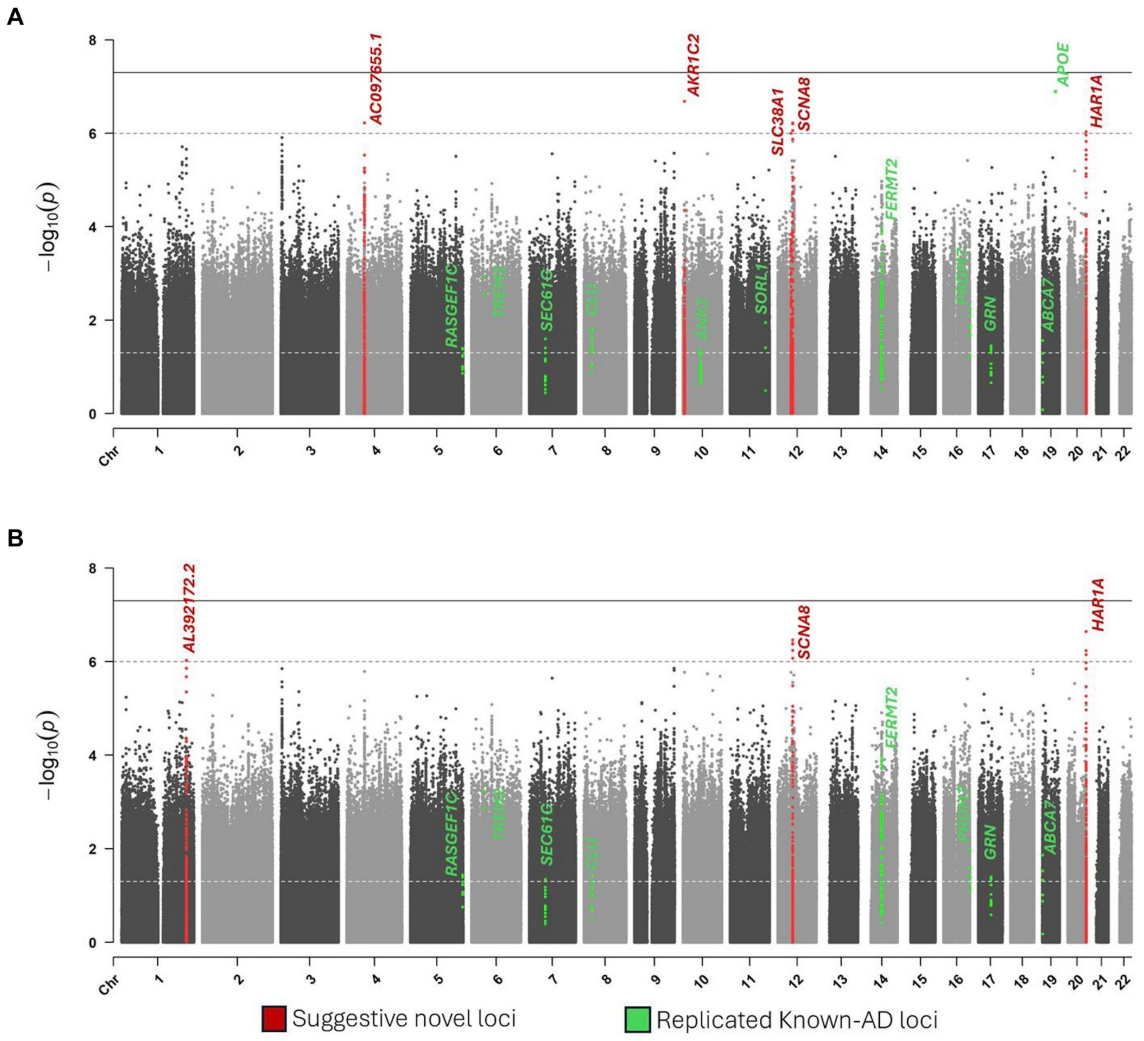
Manhattan plots of single variant analysis. **(A)** Model 1. **(B)** Model 2.

Single-variant testing replicated the *APOE* locus (*p* = 1.3 × 10^−7^) (Table 2; Figure 2). In addition to *APOE*, we replicated the same signals of ten known-AD loci (*p* ≤ 0.05): *ABCA7, ANK3, CLU, FERMT2, GRN, PRDM7, RASGEF1C, SEC61G, SORL1,* and *TREM2* (Table 2; Figure 2).

**Table 2:** Results of single variant analysis.

					Model 1		Model 2	
Closest gene	Marker	dbSNP	Reference/effect allele	AF	OR (95% CI)	*p* value	OR (95% CI)	*p* value
Novel loci								
*AL392172.2*	1:222779085	rs4240935	G/T	0.39	1.76 (1.39–2.22)	2.2 × 10^−6^	1.82 (1.43–2.31)	9.5 × 10^−7^
*AC097655.1*	4:60211881	rs11131227	A/C	0.30	0.5 (0.66–0.38)	6 × 10^−7^	0.51 (0.67–0.38)	1.6 × 10^−6^
*AKR1C2*	10:5008180	rs11252881	T/A	0.50	0.54 (0.68–0.42)	2.1 × 10^−7^	0.56 (0.71–0.44)	1.7 × 10^−6^
*SLC38A1*	12:46230329	rs11183403	A/C	0.34	1.78 (1.41–2.25)	1 × 10^−6^	1.78 (1.41–2.25)	1.7 × 10^−6^
*SCN8A*	12:51658428	rs7953996	G/A	0.20	1.98 (2.59–1.51)	6 × 10^−7^	2.03 (2.67–1.54)	4.1 × 10^−7^
*HAR1A*	20:63109757	rs112918561	T/TTG	0.16	2.04 (1.54–2.72)	9.1 × 10^−7^	2.14 (1.6–2.86)	2.3 × 10^−7^
AD-Known Loci								
*RASGEF1C*	5:180201150	rs113706587	G/A	0.11	1.41 (1.02–1.96)	0.040	1.43 (1.02–2.01)	0.036
*TREM2*	6:41161469	rs143332484	C/T	0.008	3.76 (1.58–8.95)	0.003	4.18 (1.74–10.01)	0.001
*SEC61G*	7:54873635	rs76928645	C/T	0.09	0.62 (0.41–0.94)	0.025	0.64 (0.42–0.99)	0.045
*CLU*	8:27607795	rs11787077	C/T	0.42	0.76 (0.95–0.61)	0.016	0.78 (0.99–0.62)	0.037
*ANK3*	10:60025170	rs7068231	G/T	0.46	0.8 (1–0.64)	0.048	0.8 (1.01–0.64)	0.062
*SORL1*	11:121482368	rs74685827	T/G	0.002	4.64 (1.08–19.86)	0.039	3.16 (0.85–11.78)	0.086
*FERMT2*	14:52924962	rs17125924	A/G	0.08	1.7 (1.2–2.42)	0.003	1.78 (1.24–2.56)	0.002
*PRDM7*	16:90103687	rs56407236	G/A	0.05	1.89 (1.18–3.03)	0.008	1.73 (1.07–2.81)	0.026
*GRN*	17:44352876	rs5848	C/T	0.35	1.28 (1.01–1.62)	0.043	1.28 (1.01–1.63)	0.044
*ABCA7*	19:1050421	rs115550680	A/G	0.005	3.16 (1.14–8.78)	0.027	3.62 (1.3–10.07)	0.013
*APOE*	19:44908684	rs429358	T/C	0.13	2.19 (1.64–2.93)	1.3 × 10^−7^	1.69 (0.29–10)	0.563

Allele frequency (AF) in the table reflects the effect allele frequency in the controls. The AD-Known markers are the same SNPs identified in the AF (Kunkle et al., 2021) and NHW (Bellenguez et al., 2022) GWASs.

Six novel loci reached suggestive significance at *p* ≤ 1 × 10^−6^: *AL392172.2* on chromosome 1, *AC097655.1* on chromosome 4, *AKR1C2* on chromosome 10, *SLC38A1* on chromosome 12, *SCN8A* on chromosome 12, and *HAR1A* on chromosome 20 (Table 2; Figure 2).

As a result of gene-based testing, there was no gene-wide significant region (Supplementary Table S1).

### 3.2 Pathway analysis

MAGMA gene-set analysis showed no pathway at P_bon_ < 0.05 after Bonferroni correction (18977 genes were tested). Three pathways were identified that *p* < 1 × 10^−4^, although these pathways were not significant after Bonferroni correction (Supplementary Table S2).

### 3.3 Fine mapping and replication analysis

Six novel loci in Models 1 and 2 were fine mapped using CARMA. There was no credible set generated for these regions, although these regions’ index SNPs showed the highest PIPs (Supplementary Table S3). Consequently, these SNPs garnered a larger proportion, if not the entirety, of the PIP for their respective regions with the sum of the PIPs of these regions falling short of generating a credible set.

Replication analysis in an independent Caribbean Hispanic dataset from the EFIGA study (632 AD, 270 cognitively unimpaired) showed significant associations of index SNPs in two loci: *SLC38A1* (*p* = 0.009), and *SCN8A* (*p* = 0.049). As a result of the metanalysis of the EFIGA and our PR datasets, the SLC38A1 locus neared genome-wide significance (*p* = 3×10^−7^).

### 3.4 Admixture mapping

**Figure 3:**
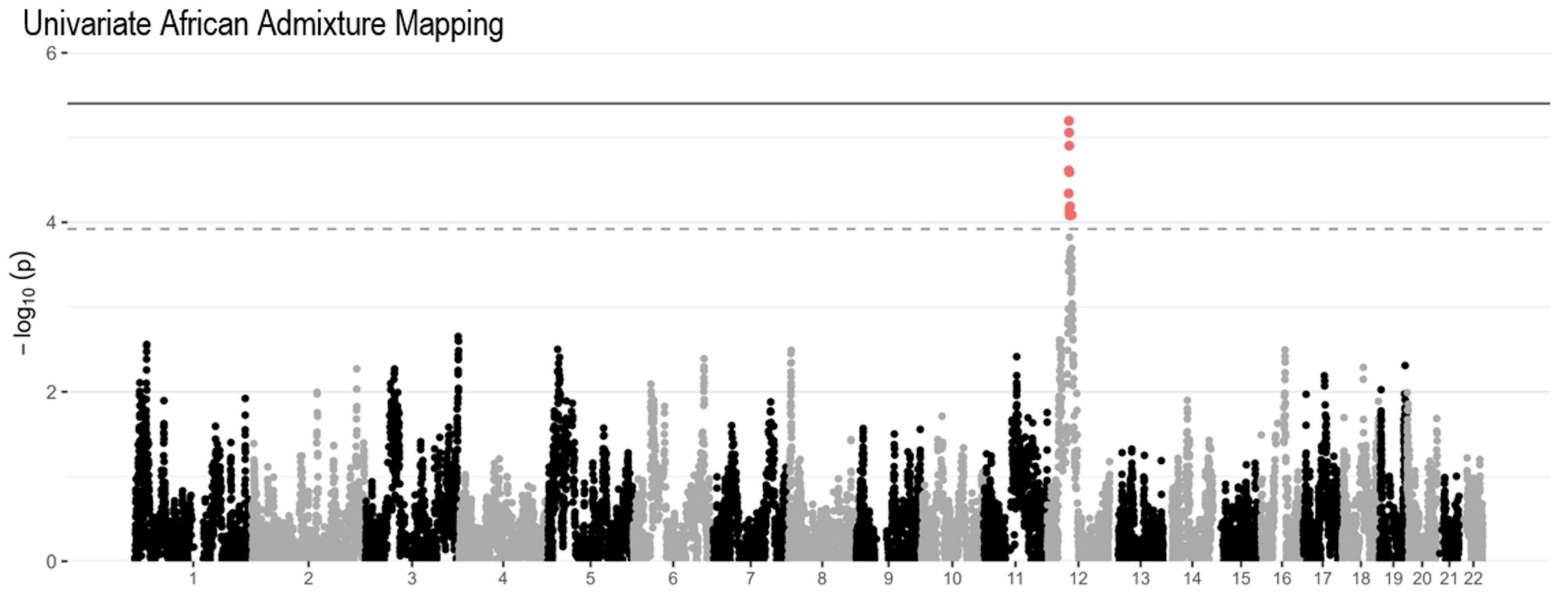
Univariate African AM Manhattan plot of PR dataset. The solid gray horizontal line represents the genome-wide significance threshold calculated in our cohort, and the dashed gray horizontal line represents the genome-wide significance threshold calculated in the previous larger Caribbean Hispanic study (Kizil et al., 2022).

An ancestral block located on chromosomes 12q13.1 (*p* = 6.3×10^−6^, Figure 3) neared genome-wide significance by Univariate African AM analysis. This region also overlapped with the *SLC38A1 and SCN8A* genes, which reached suggestive significance in the association analysis.

### 3.5 Global ancestry and ROH analysis

**Figure 4:**
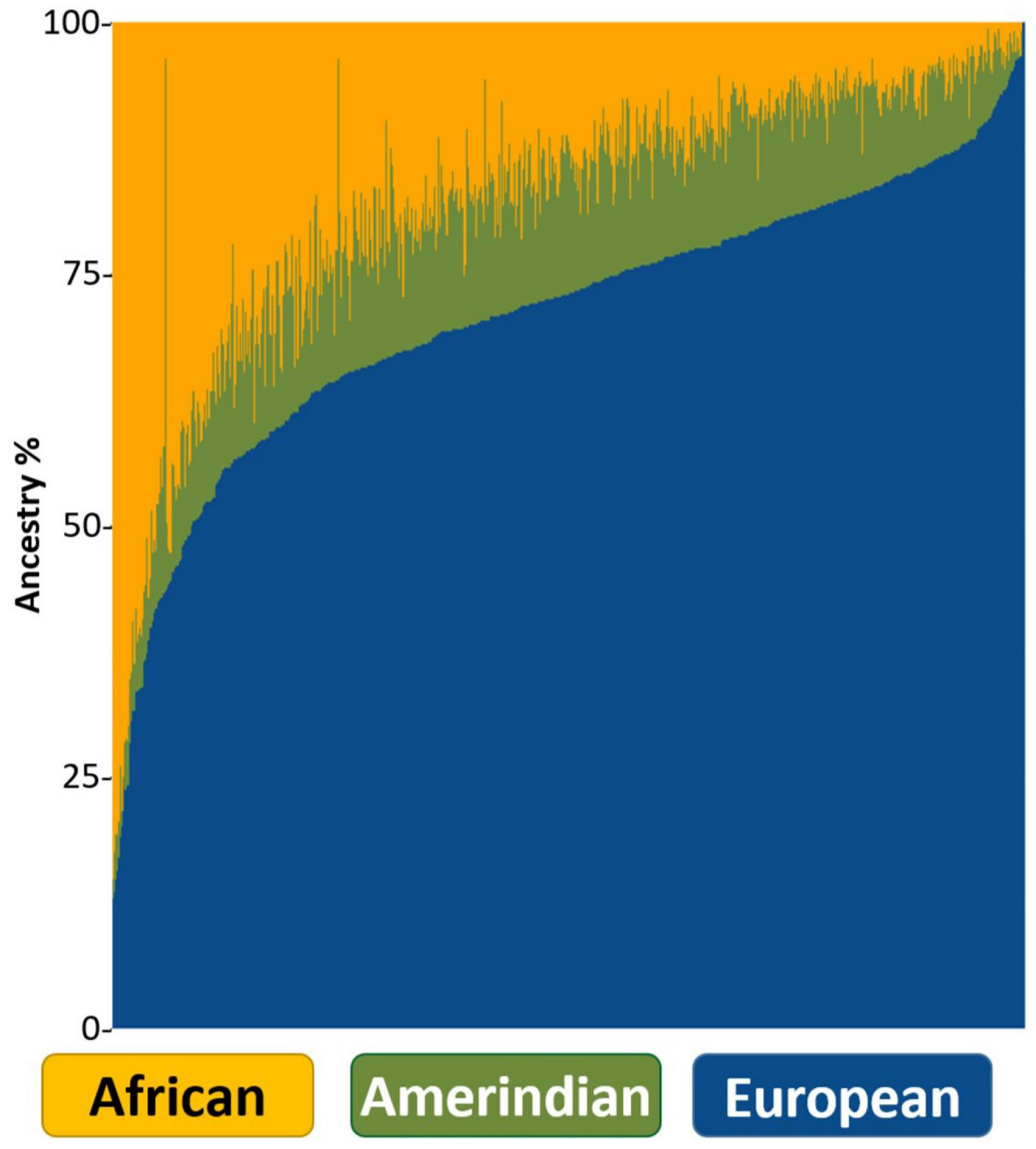
ADMIXTURE bar plot showing each individual as a vertical line and global ancestries in different colors.

Admixture analysis revealed proportions of 71% EU, 18% AF, and 11% AI in the cohort (Figure 4). Global ancestry distributions according to different health regions (Puerto Rico Department of Health, 2024) in PR showed a slight increase in the AF rate and a decrease in the EU in Zone 7 compared to the others (Supplementary Figure S1A). In addition, it was observed that the ROH length and number distributions of the participants in Zone 7 mostly overlapped with the reference individuals of African origin (Supplementary Figure S1B).

Global Burden Analysis showed that the mean size of ROHs larger than 1 MB was significantly higher in cases than in the control group (Supplementary Table S4).

### 3.6 Polygenic risk score

**Figure 5:**
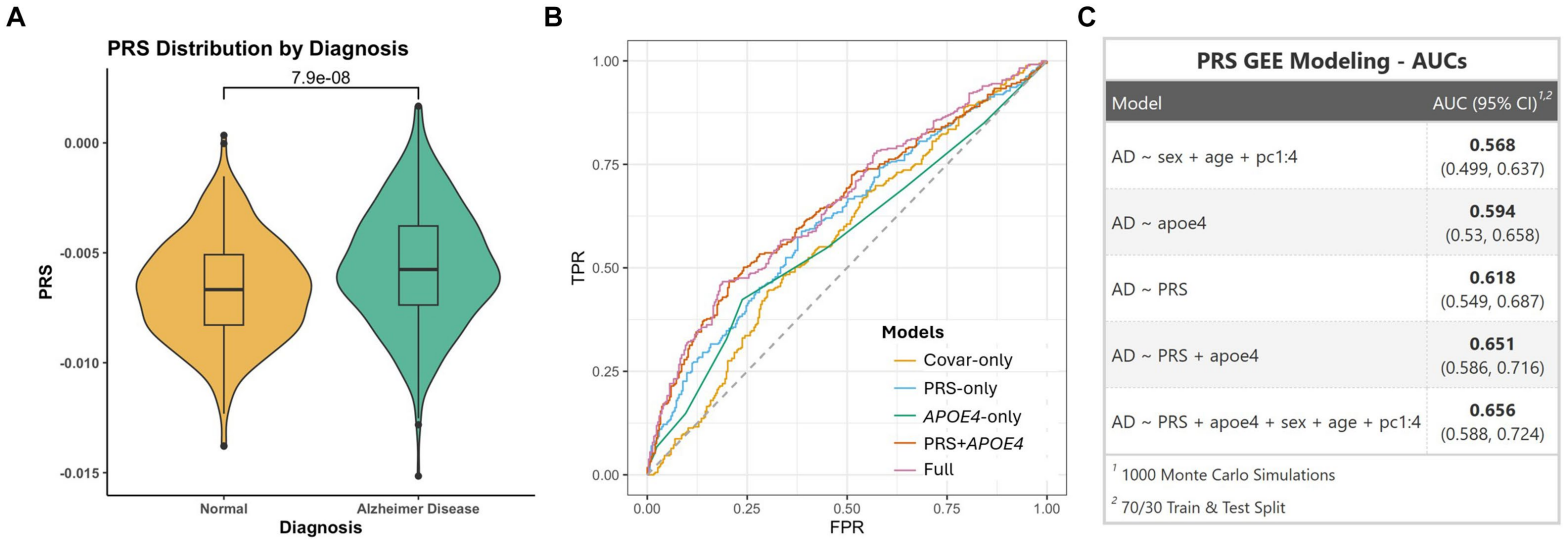
**(A)** Violin plot showing PRS distribution between AD and cognitively unimpaired individuals. **(B)** ROC curves showing different models. **(C)** Table showing AUC values for different models.

We calculated a PRS using 99 clumped SNPs (Supplementary Table S5). AUC in the PR dataset was found to be 0.62 in Model_PRS-only_ and the *t*-test showed a significant association between PRS and AD (*p* = 7.9×10^−8^) (Figure 5A). In model_*APOE ε4*-only_ and model_PRS + *APOE ε4*_, we achieved an AUC of 0.59 and 0.65, respectively. Model_Full_ showed an AUC of 0.66 (Figures 5B,C).

## 4 Discussion

Our GWAS and AM analysis identified a suggestive AD risk locus with two signals within a 5 MB region on chromosome 12: one within the *SLC38A1* gene (12q13.11) and the other within the *SCN8A* gene (12q13.13). We replicated both signals using an independent Caribbean Hispanic dataset from the EFIGA study. This region corresponds to a locus on chromosome 12q13 previously implicated in AD by linkage studies (Rogaeva et al., 1998; Yu et al., 2011; Scott et al., 2000; Pericak-Vance et al., 1997; Beecham et al., 2009). The index marker at 12q13.11, identified in this study, was found to be significant in the AF (Kunkle et al., 2021) (*p* = 0.004; OR = 1.12) and NHW (Kunkle et al., 2019) (*p* = 0.04; OR = 1.03) GWAS studies, further supporting these findings. The ancestry approach showed that the index marker has both EU and AF ancestral backgrounds and both contributing to the risk in this region. The *SLC38A1* gene is associated with ischemic brain damage (Yamada et al., 2019) and its transcription is affected by amyloid-beta peptide (Buntup et al., 2008). The *SCN8A* gene is associated with a severe developmental and epileptic encephalopathy (Ohba et al., 2014), cognitive impairment (Wagnon et al., 2017; Trudeau et al., 2006), and has a demonstrated relationship with reduced pathogenesis of AD in a mouse model study (Yuan et al., 2022). Both genes are involved in the biological process of sodium ion transport (GO [Gene Ontology]:0006814) (Aleksander et al., 2023; Ashburner et al., 2000). While the number of participants in this study is modest, the robustness of our findings at this locus is further strengthened by the replication in an independent Caribbean Hispanic cohort, ancestry-aware follow-up analysis, and supporting results from previous GWAS and linkage studies.

We replicated the *APOE e4* risk allele and additionally the same markers of the ten known AD loci (Bellenguez et al., 2022; Kunkle et al., 2021) – *ABCA7, ANK3, CLU, FERMT2, GRN, PRDM7, RASGEF1C, SEC61G, SORL1,* and *TREM2. APOE ε4* allele is the major risk factor for AD in almost all populations, but its effect differs among different ancestral populations (Farrer et al., 1997). The ε4 allele has the highest risk in East Asian populations (Liu et al., 2014), followed by Europeans, and a lower risk in AF ancestry populations (Tang et al., 1996; Tang et al., 1998; Sahota et al., 1997; Hendrie et al., 2014). The *APOE e4* odds ratio was found to be 2.19 (1.64–2.93) in our study, and although this rate was slightly above that in the recent large-scale African-American GWAS study (OR = 1.93) (Kunkle et al., 2021), it was below that found in European studies. Our result was also consistent with a study investigating the ancestral origin of *APOE e4* AD risk in PR and African American populations (Rajabli et al., 2018). Of the 10 other signals replicated by our study, 9 were identified in European studies (Bellenguez et al., 2022) and *ABCA7* (rs115550680) was identified in the recent African-American GWAS study (Kunkle et al., 2021). This is likely due to the higher proportion of EU background and the lower proportion of AF background of the PR population.

Global ancestry admixture analysis revealed proportions of 71% EU, 18% AF, and 11% AI in our cohort, which confirmed that PRs were a 3-way admixed population. Upon examining the global ancestry and ROH length/number distributions by zones, we saw that Zone 7 had a higher African ancestry background and a lower European ancestry background than the other zones. Upon closer inspection of the cities in Zone 7, we found out that individuals from Loiza city had African ancestry rates of 58%, which was higher than the cohort average. Loiza is known in PR for the rich African heritage that forms the basis of its identity. The background of this rich African heritage dates back to the African individuals who were brought to work in the sugar plantations established in the region in the 16th century (Perez, 2002).

NHW GWAS (5)-derived PRS showed a good predictive value (AUC of 0.62 in Model_PRS-only_) of AD risk in the PR population. Moreover, the AUC value of the PRS + APOE model was found to be higher (0.65). While the results provide a promising prediction value, there is potential to further optimize the PRS calculations for PR to enhance their clinical relevance. The accuracy of PRS improves when modelled using GWAS with a similar ancestral origin (Choi et al., 2020). Nonetheless, the NHW GWAS-based PRS likely showed good predictive results due to the substantial EU ancestral background among PRs. Overall, our results point to the importance of performing population-specific studies to derive PRS calculations that will yield high predictive values that are suitable for clinical use.

The poor generalizability of genetic studies across populations is well-established. To understand the myriad genetic factors that contribute to the development of AD it is important to study diverse populations which are underrepresented in genetic studies. By including diverse populations, not only can we identify factors that contribute to health disparities, but we can also fine-tune our efforts to develop effective treatments for AD. Further, by including underrepresented populations in genetic studies, higher-sensitivity risks can be calculated with methods such as PRS: more importantly, new genetic loci can be discovered, as in our study, and the biological role of known loci in different populations can be understood more clearly. Thus, a more effective approach to the prevention of AD can be achieved by initiating treatments at the preclinical stage (Andrieu et al., 2015), a timing frame when the pathophysiological mechanisms of the disease begin, decades before the clinically detectable symptoms of AD appear (Sperling et al., 2014). Including underrepresented populations such as the PR population, provides an important opportunity to evaluate the role of different ancestral backgrounds in AD, and may pave the way for more accurate prevention, early detection, and intervention of AD in this and other admixed Hispanic populations.

## Data Availability

The datasets presented in this study can be found in online repositories. The names of the repository/repositories and accession number(s) can be found at: https://dss.niagads.org/datasets/ng00067/, NG00067, https://dss.niagads.org/sample-sets/snd10031/, snd10031, https://dss.niagads.org/cohorts/puerto-rican-alzheimers-disease-initiative-pradi/, Puerto Rican Alzheimer’s Disease Initiative (PRADI).

## Footnotes

^1^ https://github.com/hihg-um/PuertoRican_GWAS
