# Supplementary material for: Genome-wide association analysis and admixture mapping in a Puerto Rican cohort supports an Alzheimer disease risk locus on chromosome 12
Source: Front Aging Neurosci. 2024 Sep 4;16:1459796. doi: 10.3389/fnagi.2024.1459796 (PMC11408238; doi:10.3389/fnagi.2024.1459796)
Supplement: Supplementary file 1 [file Data_Sheet_1.DOCX]

Supplementary Material

# Supplementary Figures and Tables

## Supplementary Figures


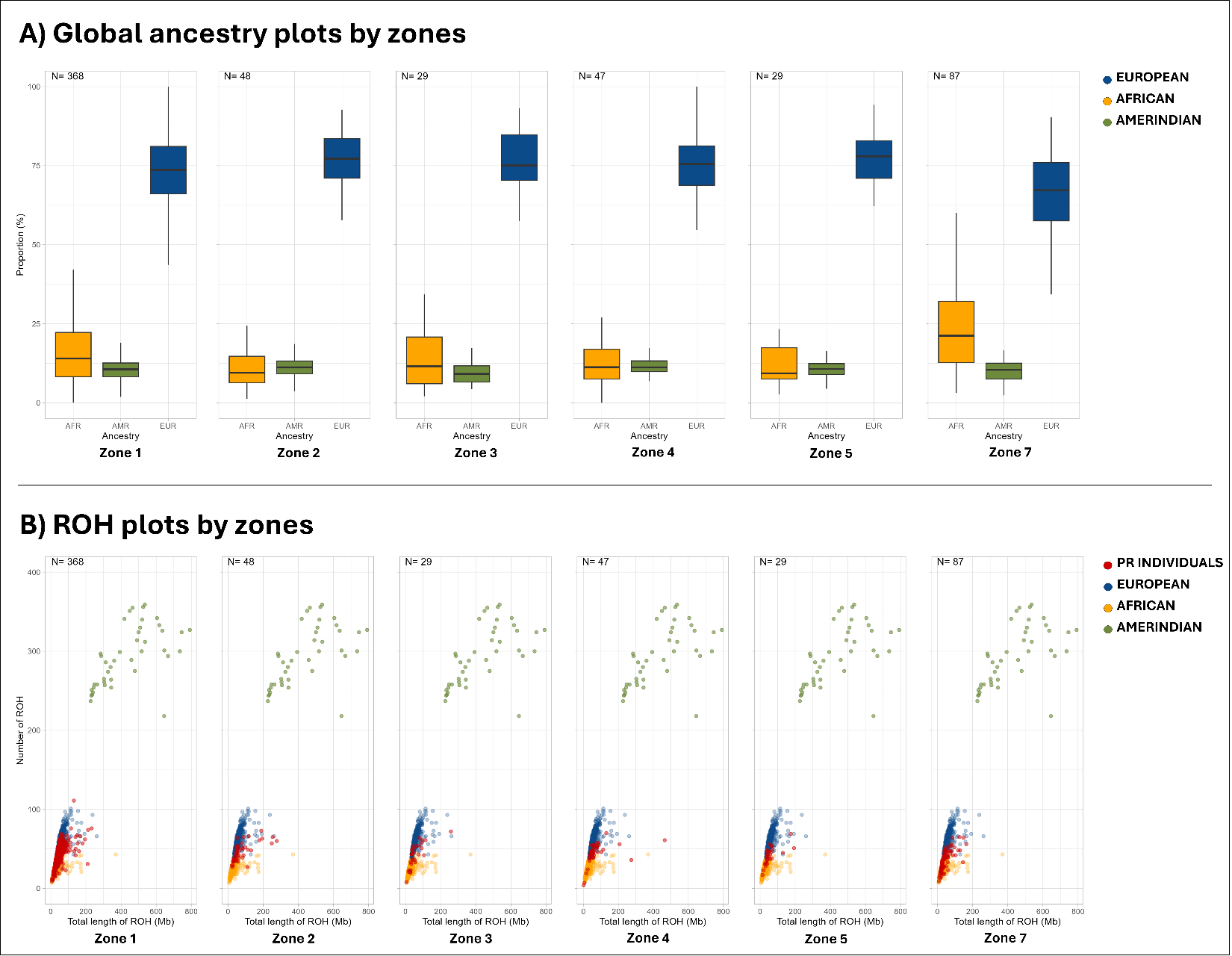


**Supplementary Figure 1.** **A)** Global ancestry proportions of individuals in PR by 6 different zones. The yellow color represents the African background, the blue represents the European background, and the green represents the Amerindian background. **B)** Distributions of total ROH length and number of ROHs in participants from six different zones in PR (represented in red). The yellow, blue, and green colors represent individuals from the HGDP and 1000G reference panel with African, European, and Amerindian backgrounds, respectively. Since there were only two participants from Zone 6, this zone was included in the figure.

## Supplementary Tables

**Supplementary Table 1.** Regions at P < 0.0001 as a result of rare variant gene-based testing for Model 1 (adjusted for sex, age, and first 4 PCs as fixed effects and GRM as a random effect), and Model 2 (also adjusted for APOE-ε4 allele dosage).

|  |  | **Region** | **Pvalue** | **Pvalue_Burden** | **Pvalue_SKAT** | **BETA_Burden** | **SE_Burden** |
| --- | --- | --- | --- | --- | --- | --- | --- |
| **Model 1** | **CADD0** | *ISL1* | 5.106E-06 | 5.349E-06 | 8.286E-06 | 0.1313922 | 0.0288735 |
|  | **CADD10** | *BEGAIN* | 5.368E-06 | 5.368E-06 | 5.368E-06 | -0.1611823 | 0.0354257 |
|  |  | *BCAT1* | 1.891E-05 | 1.891E-05 | 1.891E-05 | 0.2456142 | 0.0574218 |
|  |  | *NPM1* | 8.666E-05 | 0.2736582 | 4.715E-05 | 0.0213338 | 0.0194886 |
| **Model 2** | **CADD0** | *ISL1* | 1.557E-05 | 1.483E-05 | 2.525E-05 | 0.1258767 | 0.0290631 |
|  | **CADD10** | *BEGAIN* | 1.367E-06 | 1.367E-06 | 1.367E-06 | -0.1771946 | 0.0366882 |
|  |  | *BCAT1* | 6.06E-06 | 6.06E-06 | 6.06E-06 | 0.2670644 | 0.0590292 |

**Supplementary Table 2.** Top pathways derived from MAGMA gene-set analysis.

| **Gene-set name** | **Number of Genes** | **P value** | |
| --- | --- | --- | --- |
|  |  | **Model 1** | **Model 2** |
| Lin tumor escape from immune attack | 18 | 3.9 x 10^-5^ | 1.8 x 10^-4^ |
| Chiang liver cancer subclass polysomy7 dn | 25 | 3.4 x 10^-4^ | 7.0 x 10^-5^ |
| Gobp regulation of inflammatory response to antigenic stimulus | 13 | 1.2 x 10^-4^ | 1.7 x 10^-5^ |

**Supplementary Table 3.** PIP values ​​as a result of fine mapping of index markers of suggestive significant loci

| **Closest gene** | **Marker** | **dbSNP** | **Reference**  **/effect allele** | **PIP – Model 1** | **PIP – Model 2** |
| --- | --- | --- | --- | --- | --- |
| *AL392172.2* | 1:222779085 | rs4240935 | G/T | 0.34 | 0.38 |
| *AC097655.1* | 4:60211881 | rs11131227 | A/C | 0.22 | 0.18 |
| *AKR1C2* | 10:5008180 | rs11252881 | T/A | 0.96 | 0.79 |
| *SLC38A1* | 12:46230329 | rs11183403 | A/C | 0.86 | 0.84 |
| *SCN8A* | 12:51658428 | rs7953996 | G/A | 0.08 | 0.11 |
| *HAR1A* | 20:63109757 | rs112918561 | T/TTG | 0.18 | 0.32 |

**Supplementary Table 4.** Global Burden Measurements of ROHs in PR individuals. (Bonferroni-adjusted p-value = 0.0167)

|  | **1 Mb** | | | **2 Mb** | | | **3 Mb** | | |
| --- | --- | --- | --- | --- | --- | --- | --- | --- | --- |
|  | Cases | Controls | P value | Cases | Controls | P value | Cases | Controls | P value |
| **Total Number of ROHs** | 5177 | 4121 |  | 1517 | 1031 |  | 870 | 587 |  |
| **Number of ROHs per person** | 15.05 | 13.6 | 0.0012 | 4.74 | 3.83 | 0.0023 | 3.51 | 2.84 | 0.0113 |
| **Total size of ROHs per person, MB** | 42.74 | 32.5 | 0.0003 | 30.66 | 21.32 | 0.0024 | 33.27 | 22.63 | 0.0022 |
| **Mean ROH size per person, MB** | 2.52 | 2.23 | 0.005 | 5.21 | 4.82 | 0.0872 | 7.92 | 7.41 | 0.1296 |

**Supplementary Table 5.** List of clumped 99 SNPs used to construct the PRS.

| rs12041364 | rs6846529 | rs71519637 | rs10898437 | rs56407236 |
| --- | --- | --- | --- | --- |
| rs2070902 | rs62375397 | rs62504296 | rs10792832 | rs1323085091 |
| rs6540874 | rs113706587 | rs7341557 | rs11603136 | rs78534596 |
| rs679515 | rs113472359 | rs10780145 | rs138337950 | rs5848 |
| chr1:207664601 | rs143332484 | rs867230 | rs60228070 | rs2680700 |
| rs2404174 | rs9394766 | rs138529507 | rs11218343 | rs4292 |
| rs75836995 | rs4714447 | rs199563267 | rs11218360 | rs72618590 |
| rs17014873 | rs9349413 | rs1693551 | rs11828225 | rs72973575 |
| rs11694743 | rs1777484658 | rs34674752 | rs17125924 | rs4147910 |
| rs12989701 | rs67250450 | rs34173062 | rs12590654 | rs12151021 |
| rs34546266 | rs74504435 | rs992537601 | rs2009833 | chr19:1066626 |
| rs6733839 | rs4424195 | rs3740204 | rs183428733 | rs72975514 |
| rs6431223 | rs4727449 | rs7912495 | rs593742 | rs12462743 |
| rs7569598 | rs7384878 | rs7068231 | rs11853804 | rs1555830266 |
| rs7421448 | rs13235951 | rs6586028 | rs117618017 | rs12462098 |
| rs28459768 | rs2734895 | rs10838702 | rs1140239 | rs6014724 |
| rs61762319 | rs56402156 | rs12577383 | rs78924645 | rs2154481 |
| rs4690197 | rs11769559 | rs562028 | rs12444183 | rs2830489 |
| rs3916085 | chr8:11817388 | rs1582763 | rs147330136 | rs2830510 |
| rs875394 | rs73223431 | rs638509 | rs4485362 |  |
